# Analysis of Factors Contributing to the Increase in ^7^Be Activity Concentrations in the Atmosphere

**DOI:** 10.3390/ijerph191610128

**Published:** 2022-08-16

**Authors:** Yukinori Narazaki, Akihiro Sakoda, Naofumi Akata, Hisanori Itoh, Noriyuki Momoshima

**Affiliations:** 1 Fukuoka Institute of Health and Environmental Sciences, 39 Mukaizano, Dazaifu 818-0135, Japan; 2 Ningyo-toge Environmental Engineering Center, Japan Atomic Energy Agency, 1550 Kamisaibara, Kagamino-cho, Tomata-gun 708-0698, Japan; 3 Institute of Radiation Emergency Medicine, Hirosaki University, 66-1 Hon-cho, Hirosaki 036-8564, Japan; 4 Department of Earth and Planetary Sciences, Kyushu University, 744 Motooka, Nishi-ku, Fukuoka 819-0395, Japan; 5 Central Institute of Radioisotope Science and Safety Management, Kyushu University, 744 Motooka, Nishi-ku, Fukuoka 819-0395, Japan

**Keywords:** ^7^Be, atmosphere, increased concentration, factor analysis

## Abstract

In March 2013, increased ^7^Be activity concentrations in the atmosphere were observed for successive days in Dazaifu, western Japan. The daily ^7^Be activity concentration ranged from 0.93 to 14 mBq/m^3^, with a monthly average of 8.3 mBq/m^3^. This average was the highest among the monthly averages observed between 1999 and 2015, and higher than the monthly average over this period (4.7 mBq/m^3^) plus twice the standard deviation. Also, this exceeded the monthly average (6.0 mBq/m^3^) only for March 1999–2015 (excluding 2013, when the cosmic-ray intensity, a component producing ^7^Be, decreased). Based on the backward trajectory analysis etc. results, the inflow of air from the stratosphere and upper troposphere at high latitudes that frequently occurred in March 2013 was considered the reason for the ^7^Be activity concentration increase.

## 1. Introduction

Beryllium-7 (^7^Be) is a cosmogenic radionuclide produced by nuclear spallation reactions between oxygen or nitrogen atoms and cosmic rays in the stratosphere and the upper troposphere. The ^7^Be production amount depends on the intensity of cosmic rays entering the atmosphere and the target element types and concentrations, the former being the primary factor. The cosmic-ray intensity depends on geomagnetic latitude and altitude and is associated with solar activity [1].

Cosmic rays include high-energy radiation originating from supernova explosions and the sun. Primary cosmic rays entering the earth’s atmosphere mainly comprise of protons (more than 90%), and the rest contain nuclei, such as alpha particles [2]. The primary cosmic rays collide with air in the upper atmosphere and produce various secondary cosmic rays, among which protons and neutrons primarily undergo spallation reactions to produce the radioactive isotope ^7^Be. ^7^Be has an overwhelmingly high production rate of 1960 PBq/year [3]. The produced ^7^Be atoms are oxidized and exist in the atmosphere as solid ^7^BeO or ^7^Be(OH)_2_ adsorbed on aerosols [4,5]. ^7^Be can be quantified by non-destructive measuring of its emitting gamma rays, and it has low chemical reactivity. Therefore, it has long been used as a tracer of aerosols and chemical substances, and as an indicator to study the movement of atmosphere between the stratosphere and the troposphere.

Atmospheric ^7^Be near ground level generally seems to be originated from the upper troposphere, due to its relatively short physical half-life (53.3 days) and long removal time (1–2 years) in the stratosphere [6]. The following factors are known to produce the (seasonal) fluctuations in ^7^Be activity concentration levels at ground level [7,8,9]: cosmic-ray intensity [10,11], the exchange of atmosphere between the troposphere and stratosphere [12,13], the north–south or east–west mixing of atmosphere [14], and the frequency of precipitation [15,16].

The clarification of the global distribution, behavior and location characteristics for ^7^Be allows for the identification of the previously unknown way in which upper atmospheric particles are carried from the stratosphere and the upper troposphere to ground level in East Asia, as well as the understanding of its distribution and moving path. Long-term continuous monitoring of accurate ^7^Be activity concentrations is essential to achieve this.

^7^Be activity concentrations in the atmosphere frequently exceeded monthly average values in short periods. Interestingly, remarkably high concentrations were observed at our monitoring location during most of March 2013, resulting in the monthly mean being more than the monthly average over 17 years (1999–2015) plus twice the standard deviation. This study shares this exciting phenomenon and attempts to better understand the dynamics of ^7^Be in the atmosphere by discussing contributing factors of this unexpected increase in ^7^Be activity concentrations. It would be helpful in particular to understand air parcels descending from the stratosphere and the upper troposphere as well as climate variables affecting the transport and deposition of aerosols.

## 2. Materials and Methods

### 2.1. Aerosol Sampling and Precipitation Monitoring

Aerosol samples were collected on the rooftop (15 m above ground level, 30 m above sea level) of the Fukuoka Institute of Health and Environmental Sciences (FIHES) (33°30′ N, 130°30′ E) in Dazaifu City, Fukuoka Prefecture, Japan. FIHES is dedicated to environmental conservation and public health, including monitoring radioactive materials. The sampling was conducted from January 1999 to December 2015, and 2898 data were obtained. The samples were collected successively for one to four days before April 2011 and every day after April 2011. Several samples were missed each year due to planned power outages etc. in the facility where the sampling equipment was installed. Also, the sampling was stopped during seismic strengthening work of this facility in October (26 days) and November (19 days) 2014.

The sampling was performed by operating a high-volume air sampler (HV-1000N, 1000F or 1000R, SIBATA, Tokyo, Japan) at 1000 L/min. The sampler had quartz fiber filters (QR-100, ADVANTEC, Tokyo, Japan) with an area of 20.3 cm × 25.4 cm (effective sampling area: 414 cm^2^). Before April 2011, the sampling started, in principle, at 9:00 AM, and the total sampled air volume per sampled filter was 1440–5500 m^3^. In April 2011 and after, the sampling started at 9:00 AM and finished at 9:00 AM the next day, providing a total sampled air volume of 1440 m^3^. The sampling should provide 100% ^7^Be collection because the manufacturer guarantees that the QR-100 filter has a collection efficiency of 99.99%, with a velocity of 5 cm/s for 0.3 μm dioctyl phthalate (DOP) particles.

The precipitation amount and duration were monitored using an overturning-type rain gage (minimum detectable level: 0.5 mm) on the rooftop where the aerosol sampling was conducted. The precipitation amount from 9:00 AM to 9:00 AM the next day was defined as that for one day.

### 2.2. ^7^Be Activity Measurement

After sampling, the quartz fiber filter was folded in two, with the collecting side inwards. The folded filter was punched into six circles using a ϕ 47.5 mm punch, resulting in twelve circular filters. The punched filters were pressed and molded into a 2 mm-thick disk-like shape using a hydraulic machine. The punched filters’ cumulative area accounted for 51% of the effective sampling area.

The disk-shaped filter sample was placed into a measurement container (inner diameter 48 mm; height 30 mm), and the cylindrical acrylic resin (thickness 2 mm) and cylindrical Styrofoam were placed on the sample, followed by sealing the container cap. The ^7^Be activity in the sample was measured by gamma-ray spectrometry with a high-purity germanium detector as follows: GEM30185 (SEIKO EG&G ORTEC; relative efficiency 36%, FWHM 1.76 keV) in 1999–2008, GX4019 (CANBERRA; 46%, 1.83 keV) in 2008–2015, GX3018 (CANBERRA; 36%, 1.68 keV) in 2012–2015, and GMX40 (SEIKO EG&G ORTEC; 45%, 1.89 keV) in 2014–2015. The measurement time was approximately 80,000 s to obtain the measured activity within a 5% error. Photopeak energy of 477.6 keV gamma-ray was used for the analysis, and the detection efficiency was empirically determined using a mixed-standard source provided by the Japan Radioisotope Association (nine nuclides: ^109^Cd, ^57^Co, ^139^Ce, ^51^Cr, ^137^Cs, ^54^Mn, ^88^Y, ^85^Sr, and ^60^Co) or Eckert & Ziegler (10 nuclides: ^210^Pb, ^109^Cd, ^57^Co, ^139^Ce, ^51^Cr, ^137^Cs, ^54^Mn, ^88^Y, ^85^Sr, and ^60^Co). The ^7^Be activity concentration at the sampling date was calculated from the filter sample’s measured activity, considering its half-life (53.3 days) and the collected air volume. When the counting error was three times larger than the net counts, the activity was regarded as “not detected” (ND).

The gamma-ray measurement traceability was assured with annual comparison tests using five mixed-standard sources with different heights provided by the Japan Radioisotope Association (nine nuclides: ^109^Cd, ^57^Co, ^139^Ce, ^51^Cr, ^137^Cs, ^59^Fe, ^54^Mn, ^88^Y, and ^60^Co). The traceability was made based on the confirmation that the differences between the assigned values and our quantified values were less than 10%.

### 2.3. Data Analysis

^7^Be activity concentration was obtained daily, except for the missing measurement dates. The concentration quantified from a filter sample was assigned to the sampling dates when the daily sampling times were half a day or more. Consequently, a time series of ^7^Be data over 5948 days was obtained; in cases of ND, the ^7^Be activity concentration was considered 0. Monthly ^7^Be activity concentrations were determined by averaging all data quantified in the corresponding month. Hourly data on mass ozone concentrations were obtained from the Fukuoka Prefectural Air Pollution Monitoring System on atmospheric monitoring implemented at the same campus as this ^7^Be monitoring.

The cosmic-ray intensity was obtained from neutron counts as secondary cosmic rays observed at Oulu, Finland (65°03′ N, 25°28′ E; 0.00 m above sea level; 0.78 GV cut-off rigidity), from where 10-day backward trajectories reaching our aerosol sampling point started in many cases. The World Data Center for Cosmic Rays, Nagoya University provided the cosmic-ray data [17].

### 2.4. Backward Trajectory Analysis

Backward trajectories were traced from 1734 starting points nearby to seek the origin of air parcels reaching the FIHES, on six levels at 25 m intervals, 25–150 m above ground level, with 17 × 17 grid points at grid intervals of 5 km centered at the FIHES on one altitude surface. The model surface data of the Japanese 55-year Reanalysis [18], in which the grid and time intervals were 0.5625° and 6 h, respectively, were used in the calculation. Refer to Itoh and Narazaki [12] for the detailed calculation scheme of backward trajectories and why numerous starting points were used.

## 3. Results and Discussion

### 3.1. Monthly Variation in ^7^Be Activity Concentrations

**Figure 1:**
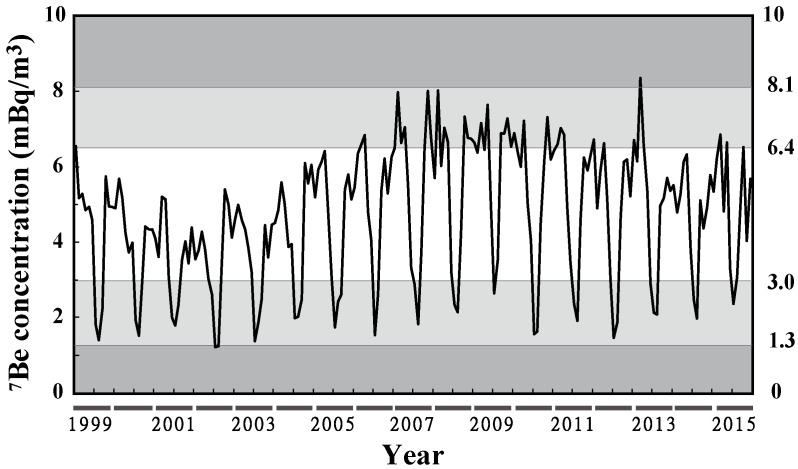
Time variation of monthly mean ^7^Be activity concentrations from 1999 to 2015. The values on the right side represent M ± SD (i.e., 3.0 and 6.4) and M ± 2SD (i.e., 1.3 and 8.1); M is the mean (4.7 mBq/m^3^), and SD is the standard deviation (1.7 mBq/m^3^).

Figure 1 shows the time variation of monthly mean ^7^Be activity concentrations from 1999 to 2015. The minimum was 1.2 mBq/m^3^ in July and August 2002, the maximum was 8.3 mBq/m^3^ in March 2013, and the mean (M) ± standard deviation (SD) was 4.7 ± 1.7 mBq/m^3^. ^7^Be activity concentrations less than 3.0 mBq/m^3^ (M–SD) accounted for 21% of all data, mostly observed in August. The concentrations were less than 1.3 mBq/m^3^ (M–2SD) in July and August 2002 only. ^7^Be activity concentrations higher than 6.4 mBq/m^3^ (M + SD) also accounted for 21% of all data, mostly observed in January–April. Of the 17 years surveyed, the concentrations were only higher than 8.1 mBq/m^3^ (M + 2SD) in March 2013.

Thus, the following section focuses on March 2013, with noticeably high ^7^Be activity concentrations, to obtain details of its daily variation. The increased concentrations observed in that month will be scrutinized from precipitation and ozone concentration (Section 3.2), cosmic-ray intensity (Section 3.3), and meteorological data (Section 3.4) viewpoints that are possible factors influencing ^7^Be activity concentrations.

### 3.2. Daily Variation in ^7^Be Activity Concentrations, Precipitation, and Ozone Concentrations

**Figure 2:**
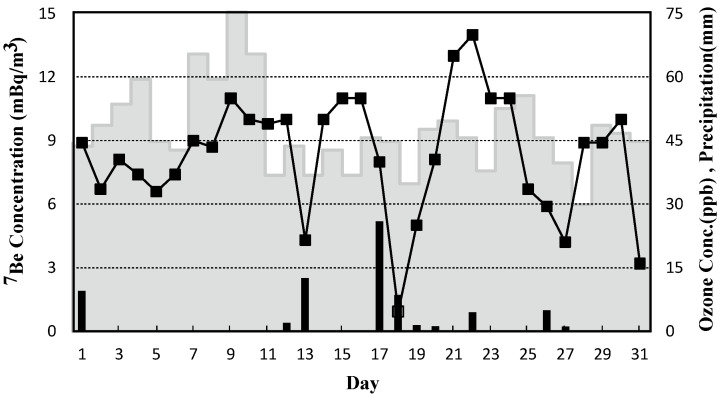
Daily variations in ^7^Be activity concentrations, daily maximum ozone concentrations in the atmosphere, and precipitation in March 2013. The ^7^Be activity concentration (■) is expressed on the left axis, and the ozone concentration (step curve) and precipitation (bar graphs) are displayed on the right.

Figure 2 shows the daily variations in ^7^Be activity concentrations, precipitation, and ozone concentrations in the atmosphere in March 2013. The ^7^Be activity concentrations were 0.93–14 mBq/m^3^. In general, precipitation and non-precipitation days tend to have lower and higher concentrations, respectively. There were 21 days without and 10 days with precipitation, and the monthly total was 69.5 mm. The ^7^Be activity concentrations remained high during the month, and the average non-precipitation days was 8.9 mBq/m^3^. There were 17 days when the daily ^7^Be activity concentrations exceeded 8.1 mBq/m^3^ (M + 2SD), accounting for 55% of March 2013 (7–12 March (8.7–11 mBq/m^3^), 14–16 (10–11 mBq/m^3^), 20–24 (8.1–14 mBq/m^3^), and 28–30 (8.9–10 mBq/m^3^)). Most non-precipitation days of March 2013 had ^7^Be activity concentrations higher than 6.0 mBq/m^3^, corresponding to the value averaged over March 1999–2015, except for 2013.

However, most days with ^7^Be activity concentrations lower than 6.0 mBq/m^3^ were rainy, decreasing on days with more than 10 mm of precipitation (13 and 17 March), and significantly decreasing on the second day of successive rainy days (13, 18 and 27 March). The highest ^7^Be activity concentration (14 mBq/m^3^) was observed on 22 March, when the precipitation was 4.5 mm. A non-precipitation day between precipitation days, i.e., 21 March, had the second-highest ^7^Be activity concentration.

Similar to ^7^Be, ozone is a chemical falling from the stratosphere. Near the earth’s surface, a known diurnal behavior occurs, showing an increase in the concentration due to photochemical production during the daytime and a decrease due to the disappearance reaction with NO and dry deposition at night. Due to its sizable diurnal variation, the discussion on the stratospheric ozone’s contribution and behavior in the atmosphere should be based on daily maximum ozone concentrations.

The daily maximum ozone concentration exceeded an environmental quality standard of 60 ppb [19] for four days from 7–10 March, and high ^7^Be activity concentrations from 8.7 to 11 mBq/m^3^ were observed on these dates. However, the daily maximum ozone concentrations during 14–16, 20–24 and 28–30 March, when ^7^Be activity concentrations were similarly high or much higher, ranged from 37 to 56 ppb within a usual variation level.

Ozone is not easily decomposed in the free troposphere’s dry atmospheric flow and can behave similarly to ^7^Be. The higher ^7^Be activity and ozone concentrations were simultaneously observed during the four days of 7–10 March, strongly suggesting that both were carried from the stratosphere to the middle troposphere. Further, the migratory anticyclone during those days promoted parcels descending from high altitudes (the mid-troposphere), indirectly carrying the stratospheric atmosphere to near ground level. On the other days of March, when ^7^Be activity concentrations were high, ozone concentrations were not significantly high due to short solar radiation time and high humidity, accelerating the ozone’s decomposition rate. In conclusion, the variation in ozone concentrations could not explain the movement of ^7^Be and its increased activity concentrations in March 2013.

### 3.3. Cosmic-Ray Intensity

**Figure 3:**
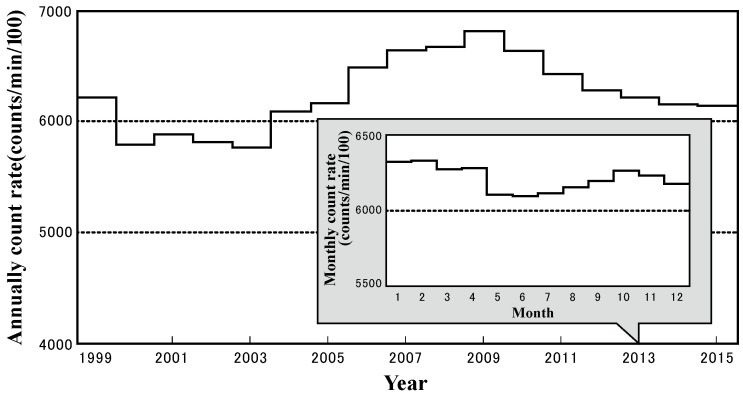
Variation in cosmic-ray intensity (neutron counts) observed at Oulu, Finland.

Figure 3 shows the variation in cosmic-ray intensity, a contributor to ^7^Be production in the atmosphere. Neutron counts of secondary cosmic rays observed at Oulu, Finland, were used to express the cosmic-ray intensity, which has been in a declining variation from 1999 to 2003, and then started to increase and reach the maximum in 2009 followed by the declining variation.

The cosmic-ray intensity depends on solar activity levels [20,21]. Due to the earth’s magnetic field structure, cosmic-ray intensities are stronger at higher latitudes and greatest in the polar regions, but smallest near the equator where the magnetic field lines are close to horizontal. Therefore, higher ^7^Be activity concentrations can be observed in the air parcels from high latitudes.

If both ^7^Be activity concentration and cosmic-ray intensity variations occur in the same way, they can be considered as being synchronized. The ^7^Be activity concentration was the lowest in July and August 2002 because the cosmic-ray intensity was the lowest, and the ^7^Be activity concentration in summer was lower than in other seasons. However, the monthly mean ^7^Be activity concentration in March 2013 was the highest in all observation periods (1999–2015), although the cosmic-ray intensity was on a declining trend in 2013 and also insignificantly high in March 2013. Furthermore, X-class solar flares were not observed during this month, and no special days occurred when daily cosmic-ray intensities were significantly high. Therefore, the cosmic-ray intensity did not contribute to the increased ^7^Be activity concentrations in March 2013.

### 3.4. Meteorological Data

Approximately 68% of the total ^7^Be amount is present in the stratosphere, and the rest is in the upper troposphere [22]. For high ^7^Be activity concentrations near the earth’s surface, air parcels with high ^7^Be activity concentrations must be rapidly transported from the stratosphere and upper troposphere, where ^7^Be is generated, to the ground surface. Itoh and Narazaki [12] found that this fast descent route is associated with tropopause folds in high latitudes, mid-tropospheric disturbances transporting air parcels from high to mid-latitudes, and disturbances by which parcels descend to the surface. Thus, we analyzed the frequency of tropopause-folding and the backward trajectory from the starting points near the FIHES.

1. Frequency of tropopause folds in high latitudes

**Figure 4:**
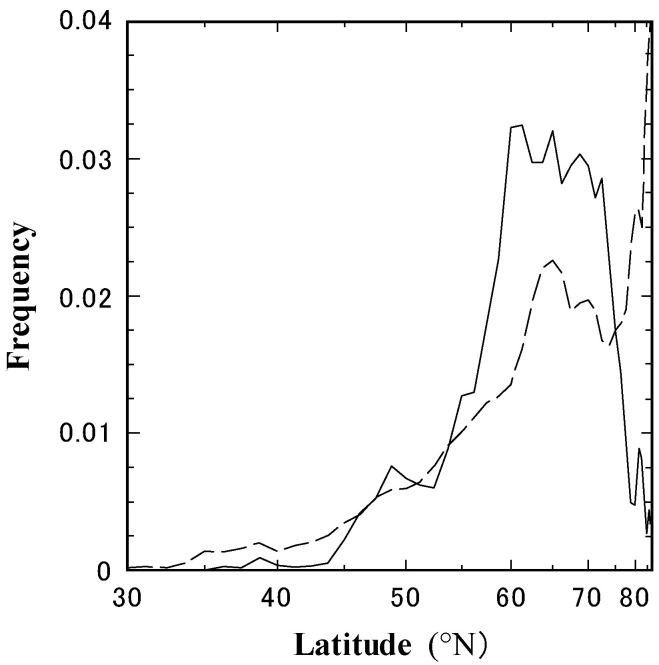
Comparison of the tropopause folds’ frequency between 30° W and 120° E in March. The solid line indicates the frequency in March 2013, and the dashed line shows the average frequency in March in the other years (1999–2015, except for 2013).

The frequency of tropopause folds between 30° W and 120° E was examined using isentropic surface data with 1.25° grid intervals. The tropopause fold is defined as the state that the surface with potential vorticity of 3 PVU hangs below 6000 m. Figure 4 compares the frequency of tropopause folds in Eurasia in March 2013 with that in the other years. Overall, tropopause fold frequencies are higher at higher latitudes. However, in March 2013, tropopause folds frequently occurred at 60°–70° N, corresponding to the originating area of high ^7^Be activity concentrations traced by backward trajectories.

2. Backward trajectory analysis

**Figure 5:**
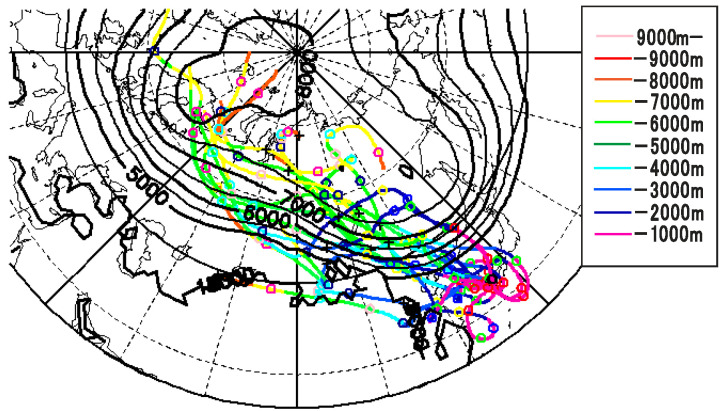
The backward trajectories reaching the top 15 highest altitudes from Dazaifu to the highest altitudes. The colors of the lines represent the altitude, with ○ and × symbols showing the positions of one and five-day intervals, respectively. The background contours indicate the 300 K isentropic surface (m) averaged in March 2013 (contours are not shown in low latitudes because isentropic surfaces intersect the earth’s surface).

Backward trajectories were traced for 10 days starting near the FIHES at 00:00 UTC each day in March 2013 (i.e., 31 days). Figure 5 three-dimensionally shows the trajectories reaching the top 15 highest altitudes (colors show the altitude). Many came from high latitudes in central Asia, with several exceptions. The comparison with the monthly averaged 300 K isentropic height indicated that the trajectories drastically dropped until reaching East Asia, then arriving at Dazaifu. Although each day’s patterns differed from the average, patterns similar to the average were dominant in this month. Therefore, air parcels with high ^7^Be activity concentrations were effectively transported to Dazaifu from high latitudes and altitudes.

**Figure 6:**
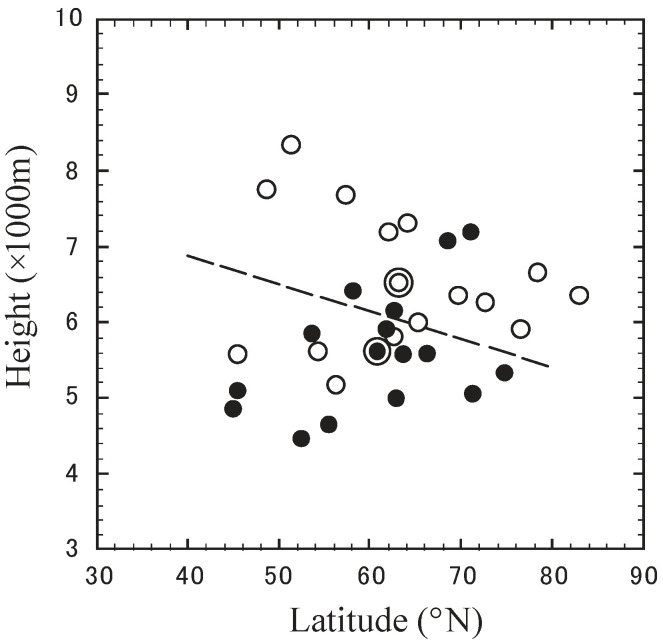
The 15 highest positions in the height–latitude section for trajectories reaching the top 15 highest altitudes among all trajectories. The symbols ○ and ● show positions in March 2013 and 2014, respectively. The double circles (◎ and ⦿) indicate the averages for the two periods, and the dashed line represents the line perpendicular to the center of the two averages.

Figure 6 plots the 15 highest altitudes in the height–latitude section among all trajectories in March 2013, with those in March 2014 as a reference plot. Only the top 15 highest cases were selected because the plot of all 31 days shows only slight differences between the two periods. A striking difference in these averaged positions can be recognized between March 2013 (6532 m, 63.2° N) and March 2014 (5614 m, 60.8° N). The former is mainly distributed in the dashed line’s upper-right area (higher altitudes and latitudes). The difference between the two average positions has a significance level of 0.05. Also, the height (918 m) and latitude (2.4°) differences are significant; however, the difference in the 31-day averages is insignificant.

In conclusion, the increase in ^7^Be activity concentrations in March 2013 can be attributed to frequent tropopause-folding events, making it easier for the atmosphere to carry ^7^Be from the stratosphere and upper troposphere to Dazaifu and the disturbance making air parcels descend to ground level.

On 21 and 22 March 2013, the observed ^7^Be activity concentrations were 13 mBq/m^3^ and 14 mBq/m^3^, respectively, corresponding to approximately 70% in the upper troposphere, as reported in the aircraft survey of Dutkiewicz and Husain [23] (19.2 mBq/m^3^ in the upper troposphere). Therefore, the increased levels of ^7^Be activity concentrations seen in our monitoring are directly linked with the upper troposphere or stratosphere, i.e., rapid descent of atmospheric flow.

## 4. Conclusions

Atmospheric ^7^Be activity concentration monitoring was implemented in Dazaifu (western Japan) for 17 years (1999–2015), indicating its remarkably high concentrations in March 2013, with the highest monthly mean. This study discussed factors affecting the considerable increase in ^7^Be activity concentrations during this month.

- Similar to the increased ozone concentrations that can be used as an analog of ^7^Be, increased ^7^Be activity concentrations were sometimes observed due to specific meteorological conditions; the inflow of air from the stratosphere or upper troposphere to the ground surface.
- The neutron counts that can produce ^7^Be in the atmosphere were insignificantly high during March 2013, indicating that cosmic rays did not play a key role.
- Air parcels frequently reached Dazaifu after migrating south from the high latitudes of Central Asia, with a remarkable drop in altitude going to East Asia.
- Factors affecting ^7^Be production were insignificant on high ^7^Be, but meteorological conditions affecting the airflow from the stratosphere and upper troposphere to the ground surface caused high ^7^Be activity concentrations in March 2013.

## Data Availability

Not applicable.
